# Molecular marker for characterization of traditional and hybrid derivatives of *Eleusine coracana* (L.) using ISSR marker

**DOI:** 10.1186/s43141-021-00277-1

**Published:** 2021-11-26

**Authors:** Jayalakshmi Venkatesan, Vasuki Ramu, Thilaga Sethuraman, Chandrasekaran Sivagnanam, Ganesh Doss

**Affiliations:** 1 Department of Plant Biotechnology, School of Biotechnology, Madurai Kamaraj University, Palkalai Nagar, Madurai, Tamil Nadu 625021 India; 2 Department of Plant Science, School of Biological Sciences, Madurai Kamaraj University, Palkalai Nagar, Madurai, Tamil Nadu 625021 India

**Keywords:** Finger millet, Genetic diversity, ISSR, Polymorphism

## Abstract

**Background:**

Finger millet is the most important food grain in the world for its nutritional benefits. Finger millet is genetically and geographically diverse and widely spread in the African and Asian sub-continent. Therefore, the present study was undertaken to analyze the genetic diversity using ISSR genetic markers using 15 ISSR primers.

**Results:**

About 23 genotypes of widely cultivated finger millet cultivars of economically important ones were characterized and the ISSR markers were critically analyzed for their performance with parameters such as polymorphic information content (PIC), effective multiplex ratio (EMR), marker index (MI), and resolving power (RP). In this study, 175 loci were scored across the 23 cultivars of finger millet, and out of these 173 loci (98%) were polymorphic, revealing the suitability of these loci for genetic diversity analysis with ISSR marker. The average number of polymorphic loci per primer was 11.50 with varying sizes from 100 bp to 2500 bp. ISSR primers that showed higher polymorphism were found to have higher EMR and MI values up to 15.30 and 13.44, respectively.

**Conclusion:**

High degree of polymorphism supported with distinct differences of all the marker parameters revealed the suitability of ISSR markers for determining the genotypic differences based on ISSR markers among the 23 genotypes of finger millet. The possible application of the ISSR marker in the conservation and management of finger millet genetic resources is discussed.

## Background

Finger millet is one of the most important food crops in Africa and Asia [1]. Globally finger millet occupies 12% in more than 25 countries in Asia and Africa [2]. Finger millet germplasm and its derivatives are said to be extremely tolerant to drought and salt stress [3]. Finger millet is well known to be one of the potential crops to alleviate the problem of nutritional deficiency being faced worldwide [4]. Considering the importance of this crop, the need for understanding the evolutionary process and conservation by understanding the genetic diversity and population genetic structure of finger millet was stressed by [5]. Molecular markers are demonstrated as an authentic tool to identify the genetic diversity and interpretation of phylogenetic relationships within and among the crop species. Crop improvement through molecular breeding can be exploited by genetic diversity assessment and population structure analysis using various DNA markers [6, 7].

Understanding the genetic diversity of finger millet is one of the vital requirements for crop improvement [8]. Genetic distance or genetic similarity are the most important parameters employed to calculate the genetic diversity of the crop plants [9]. Conventional breeding is one of the challenging issues in crop improvement of finger millet owing to its tiny flower with several other limiting factors [10]. Therefore, DNA markers become very handy to understand the genetic variability concerning useful agronomic traits for utilization in crop improvement programs and to develop viable strategies for molecular breeding [11]. In a large number of genotypes, morphological variation is characterized and linked to specific DNA markers for assessing genetic diversity [12]. Traditional morphological markers are highly useful but this can be efficiently validated by molecular markers for precise identification and placement of such genotypes in more appropriate positions based on their genetic relationships [13]. ISSR markers are proven to be the most effective tool for genetic diversity analysis owing to low cost, simplicity, reproducibility, and no prior knowledge is required. Due to the high degree of polymorphic nature, ISSR markers are extensively used [14]. Hence, the present study is to produce genetic fingerprinting of certain traditional and commercial genotypes of finger millet using ISSR markers for application in genetic improvement of finger millet.

## Methods

### Plant material and DNA isolation

Plant materials were collected from three different authentic sources through Sahaja samrudha, DHAN Foundation, and Gandhi Krishi Vignan Kendra, Bangalore, India (Table 1) Genomic DNA was extracted according to the protocol of [15] with a slight modification in extraction buffer by the addition of 0.5% SDS.

**Table 1:** Location of finger millet genotypes

Sample	Latitude(E)	Longitude (N)	Location
Biligidda	77^○^08′43.0″	12^○^00′04.1″	Biligirirangana betta
Gidda	78^○^09′48.2″	13^○^08′24.8′′	Kolar
Ibbani	78^○^04′28.1′′	13^○^14′10.0′′	Haleri
Iyan	77^○^01′59.3′′	13^○^31′05.2′′	Bhahujanahalli
Jagaluru	78^○^10′42.9′′	13^○^14′13.8′′	Hosahalli
Kappu thene	77^○^01′20.3′′	13^○^23′23.0′′	Echalakunti
Malali	77^○^03′44.5′′	13^○^24′49.6′′	Tumkur
Nachini	76^○^57′58.6′′	13^○^25′10.0′′	Nandihalli
White	75^○^19′43.5′′	14^○^47′44.2′′	Devihosur
Unde	76^○^24′50.8′′	13^○^36′19.6′′	Chitradurga
Kembu	77^○^47′14.8′′	12^○^31′22.4′′	Denkanikottai
Pichikatty	78^○^21′59.7′′	11^○^21′05.9′′	Thiruppulinadu
Saravathy	77^○^56′29.4′′	12^○^27′44.3′′	Panchapalli
Pilikatty	77^○^43′11.0′′	12^○^21′38.2′′	Anchetty
Saradha	77^○^57′38.1′′	12^○^30′23.4′′	Balaguli
Rakali sivali	78^○^19′11.7′′	11^○^19′25.3′′	Bailnadu
GPU 26	77^○^34′45.4′′	13^○^04′41.7′′	University of Agricultural Sciences, GKVK
GPU 28	77^○^34′45.4′′	13^○^04′41.7′′	University of Agricultural Sciences, GKVK
GPU 45	77^○^34′45.4′′	13^○^04′41.7′′	University of Agricultural Sciences, GKVK
GPU 48	77^○^34′45.4′′	13^○^04′41.7′′	University of Agricultural Sciences, GKVK
GPU 66	77^○^34′45.4′′	13^○^04′41.7′′	University of Agricultural Sciences, GKVK
GPU 67	77^○^34′45.4′′	13^○^04′41.7′′	University of Agricultural Sciences, GKVK
ML365	77^○^34′45.4′′	13^○^04′41.7′′	University of Agricultural Sciences, GKVK

### Screening of ISSR primers

Genomic DNA of all the genotypes of finger millets was amplified with 15 ISSR primers (Sigma-Aldrich) and assessed for their reproducibility, scorability, and ability to distinguish the selected genotypes (Table 2). The ISSR primers were selected based on the previous reports [16, 17] and used for the screening.

**Table 2:** Pairwise score of PCR amplification product of ISSR markers

Primer Name	Primer code	Total loci	Size range (bp)	Polymorphic loci	% polymorphism
ISSR 1	UBC-810(GA) _8_ T	12	250–1500	12	100
ISSR 2	UBC-811(GA) _8_ C	09	300–1200	09	100
ISSR 3	UBC-826(AC) _8_ C	12	500–2500	12	100
ISSR 4	UBC-834(AG) _8_ YT	13	200–2000	13	100
ISSR 5	UBC-835(AG) _8_ YC	14	200–1000	13	92
ISSR 6	UBC-836(AG) _8_ YA	09	250–1200	09	100
ISSR 7	UBC-840(GA) _8_ YT	09	300–2500	09	100
ISSR 8	UBC-841(GA) _8_ YC	15	300–2000	15	100
ISSR 9	UBC-842 (GA) _8_ YG	17	100–1500	18	94
ISSR 10	UBC-855 (AC) _8_ YT	07	600–1500	07	100
ISSR 11	LC-46(AC) _8_ G	14	300–2000	14	100
ISSR 12	LC-49(TG) _6_ TCTGA	12	300–2000	12	100
ISSR 13	LC-6(AG) _8_ T	11	200–1000	11	100
ISSR 14	LC-16(CT) _8_ G	10	300–2000	10	100
ISSR 15	LC-14(GA) _8_	10	300–1500	10	100

### Development of ISSR markers

Fifteen ISSR primers that shown consistent results during the previous experiment were selected for optimization of PCR condition. A factorial experiment with varying concentrations of genomic DNA (25, 30, 40, 50 ng/μl) and *Taq* DNA Polymerase (3U) was performed in different PCR cycles. The DNA samples were amplified using ISSR primers having ~ 17 bp in length with a GC content of about 48 %. PCR amplification was performed in 30 μl containing [3.0 μl of 10× *Taq* Buffer, 3.0 μl of 25 mM of MgCl_2_, 0.6 μl of 10 mM dNTP mix, 0.6 μl of 5 U of *Taq* DNA Polymerase and 0.6 μl of 10 pmol/μl of primers].

Concentrations for MgCl_2_, dNTPs, DNA polymerase, and primers, along with the annealing temperature were optimized. PCR amplification was performed on a thermal cycler (Prima–96 PLUS, HiMedia, Mumbai, India) programmed for 3 minutes at 95°C, followed by 45 cycles of 1 min at 94 °C, 1.30 min at 55 °C, 2 min at 72 °C and final stage of 10 min at 72 °C. The amplified PCR products were resolved on 1.7% agarose gel using 100 ml of 1× TAE buffer with 5 μl of EtBr and were visualized under a short wavelength UV transilluminator and documented using gel doc (Bio-Rad Laboratories Inc., 1000, USA).

### Data analysis

Data was generated based on the triplicate amplification of each arbitrary primer with consistent bands. All the finger millet samples were compared based on the banding pattern of a specific primer and cultivar specific primers were analyzed by the presence and absence of bands which were scored as ‘1’ and ‘0’ respectively. The binary data generated was used to calculate primer banding characteristics such as the number of scored bands (NSB), number of polymorphic bands (NPB), and percentage of polymorphic bands (PPB). To determine the efficiency of ISSR markers and to estimate the genetic divergence of the selected finger millet genotypes, the performance of the markers was analyzed using four parameters: (1) polymorphic information content (PIC), (2) effective multiplex ratio (EMR), (3) marker index (MI), and (4) resolving power (RP).

The method adopted by [18] was followed for calculating the PIC value of each locus by the following formula PIC_i =_ 2fi (1 − f_i_). Where PIC_i_ is the PIC of the band ‘i’, and ‘f_i_’ is the frequency of the amplified fragment present and ‘1 − f_i_’ is the frequency of non-amplified fragment absent. The frequency was calculated as the ratio between the number of amplified band set of each locus and the total number of accessions. For each primer, PIC was calculated based on the average of all loci of each primer and it is equivalent to the gene diversity.

EMR was calculated as the total number of polymorphic loci of each primer multiplied by the proportion of polymorphic loci per their total number by following the formula EMR = np(np/n). The number of polymorphic loci was denoted by “np” and the total loci number was denoted by “n”. DNA markers are more efficient when the EMR value was higher.

The marker index (MI) is a statistical tool to estimate the total utility of the marker system which can be calculated to characterize the capacity of each primer to detect polymorphic loci among the genotypes [19] based on the formula

$$\mathrm{MI}=\mathrm{EMR}\times \mathrm{PIC}$$

Where EMR (effective multiplex ratio) = *n* × *β*, where *n* is the average number of fragments amplified by accession to a specific system marker (multiplex ratio) and *β* is estimated from the number of polymorphic loci (PB) and the number of non-polymorphic loci (MB); Thus, *β* = PB/ (PB + MB) or EMR = np(np/n) [20].

The resolving power (RP) of each primer was calculated by the following formula proposed by [21].

$$\mathrm{RP}={\Sigma \mathrm{I}}_{\mathrm{b},}$$

Where *I*_b_ represents the informative fragments. The *I*_b_ can be represented on a scale of 0/1 by the following formula

$${I}_b=1-\left(2\times \left|0.5-{p}_i\right|\right)$$

Whereas the proportion of accessions containing the *i*th band was denoted by “*p*_*i”*_. To investigate the genotypic variability among finger millet, genotypes, 15 ISSR primers were tested for estimating genetic variability. Two main aspects were evaluated: marker informativeness (polymorphism and overall efficiency of informative band detection) and marker performance (overall efficacy of primer set used in determining polymorphism level, genetic diversity, and discriminatory power).

### Correlation, data scoring, and polymorphism analysis

Correlation analysis is one of the most commonly used statistical methods to determine the strength of the relationship of different ISSR markers among 23 genotypes of finger millet. The strength of the correlation coefficient can be measured based on the magnitude and direction of association between different markers. The magnitude of the correlation coefficient can be high or low whereas the direction of correlation coefficient ‘r’ can vary from − 1 to + 1 shows perfect positive (+ 1) or perfect negative (− 1) correlation and correlation coefficient with 0 shows no correlation. The correlation coefficient values were correlated among different ISSR markers. The relationship between the markers was determined if the correlation coefficient value of one ISSR marker affects or influence the value of other ISSR marker. If the correlation coefficient of one ISSR marker increases along with another ISSR marker is said to be positively correlated. If the correlation coefficient of one ISSR marker increases which affects the other ISSR marker results in decreased coefficient value is said to be negatively correlated [22]. Correlation is very small and negligible if the *r* value ≤ 0.1, low or weak correlation was observed in the range of 0.2 to 0.3, correlation level was moderate between 0.4 and 0.6, highly correlated values were in the range of 0.7 to 0.8, very highly positively correlated values were in the range of 0.9 to 1.0 whereas the above range of values in ‘− ‘sign was negatively correlated.

Data were scored based on the reproducibility of each amplicon. Data matrix were assembled and consensus profiles of ISSR marker were substantiated based on the presence (1) and absence (0) of amplicons. Standard marker analysis was performed to improve the discriminating power, informativeness and levels of polymorphism of ISSR marker using GenAlex software [23]. We estimated the following parameters to evaluate the efficiency of marker: no. of different alleles (*Na*), no. of effective alleles (*Ne*), Shannon’s information index (*I*), observed heterozygosity (*Ho*), expected heterozygosity (*He*), and unbiased expected heterozygosity (*uHe*) (Table 3).

**Table 3:** Marker parameters like levels of effective alleles, Shannon’s information index, observed heterozygosity, expected heterozygosity, and unbiased expected heterozygosity value for 23 finger millet varieties

Primer code	N	Na	Ne	I	Ho	He	uHe
UBC-810(GA) _8_ T	1.333	1.333	1.333	0.462	0.667	0.333	0.533
UBC-811(GA) _8_ C	0.000	0.000	0.000	0.000	0.000	0.000	0.000
UBC-826(AC) _8_ C	0.000	0.000	0.000	0.000	0.000	0.000	0.000
UBC-834(AG) _8_ YT	2.667	2.000	1.709	0.671	0.667	0.406	0.464
UBC-835(AG) _8_ YC	1.333	1.000	0.821	0.325	0.333	0.198	0.226
UBC-836(AG) _8_ YA	0.000	0.000	0.000	0.000	0.000	0.000	0.000
UBC-840(GA) _8_ YT	0.000	0.000	0.000	0.000	0.000	0.000	0.000
UBC-841(GA) _8_ YC	1.000	1.667	1.556	0.578	0.667	0.375	0.611
UBC-842 (GA) _8_ YG	3.667	2.667	2.433	0.921	1.000	0.582	0.698
UBC-855 (AC) _8_ YT	0.000	0.000	0.000	0.000	0.000	0.000	0.000
LC-46(AC) _8_ G	1.333	2.000	2.000	0.693	1.000	0.500	0.889
LC-49(TG) _6_ TCTGA	0.000	0.000	0.000	0.000	0.000	0.000	0.000
LC-6(AG) _8_ T	0.000	0.000	0.000	0.000	0.000	0.000	0.000
LC-16(CT) _8_ G	0.000	0.000	0.000	0.000	0.000	0.000	0.000
LC-14(GA) _8_	0.000	0.000	0.000	0.000	0.000	0.000	0.000

*Na* no. of different alleles, *Ne* no. of effective alleles, *I* Shannon’s information index, *Ho* observed heterozygosity, *He* expected heterozygosity, *uHe* unbiased expected heterozygosity

To infer the pattern of genetic structure among finger millet varieties, non-spatial Bayesian clustering method was employed using STRUCTURE 2.2.3 [24, 25]. To estimate the best number of clusters, ten independent simulations were achieved per number of sub-groups *K* (100 runs of *K* = 1–10). The output of STRUCTURE was visualized using Structure harvester program web v 0.6.94 to find the best *K* value [26]. The highest average of the estimated ln probability score was obtained based on the ideal K number that shows the lowest variance for each run. The structure output was colored in bar plots according to the maximum likelihood log with the lowest variation of the K number of sub-groups.

### Reproducibility of amplification patterns

DNA amplification with each ISSR primer was repeated at least thrice to ensure reproducibility and scorable bands for each primer were ensured before analysis. Only clear and consistent bands were considered for further analysis.

## Results

**Fig. 1:**
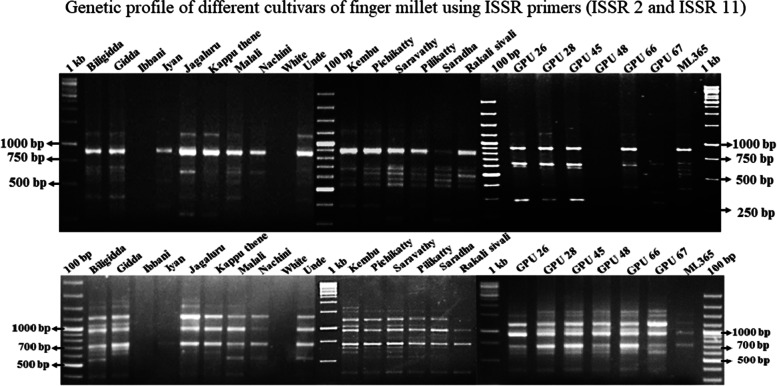
Genetic profile of different cultivars of finger millet using ISSR primers (ISSR 2 and ISSR 11)

To develop an ISSR marker for traditional and hybrid derivatives of finger millet among the various cultivars of finger millet, we tested 15 ISSR primers which revealed reproducible polymorphic patterns and were used for their performances. We have analyzed whether the ISSR primers used were ideal for use for estimating the genetic variability of the selected finger millet cultivars (Fig. 1). Based on the polymorphic information, parameters such as PIC, EMR, MI, and RP were critically analyzed for utilizing these ISSR primers for genetic variability as well as to develop cultivar-specific ISSR markers for germplasm management.

### Marker informativeness

A total of 175 ISSR loci were amplified across 23 cultivars of finger millet selected in our study. Most of the PCR products were in the size range of 100 bp to 2500 bp with the mean value of 11.6 loci per primer (Table 4). Out of 175 loci, 173 (98.8%) were found to be polymorphic and only two loci (1.2%) were monomorphic. The frequency of polymorphic loci was varied from primer to primer. The highest frequency of polymorphic loci was observed in ISSR 9 with 18 polymorphic loci followed by ISSR 8 with 15 polymorphic loci and ISSR 11 with 14 polymorphic loci. Most of the primers were found to have polymorphic loci ranging from 9 to 13. Some of the cultivars shown primer-dependent ISSR genetic fingerprints in our study.

**Table 4:** Marker parameters calculated for selected ISSR primers

Primer code	PIC	EMR	MI	RP
UBC-810(GA) _8_ T	0.32	12.00	3.84	10.80
UBC-811(GA) _8_ C	0.40	9.00	3.60	8.60
UBC-826(AC) _8_ C	0.31	12.00	3.72	7.00
UBC-834(AG) _8_ YT	0.34	13.00	4.42	14.28
UBC-835(AG) _8_ YC	0.34	12.06	4.10	9.28
UBC-836(AG) _8_ YA	0.20	9.00	1.80	9.56
UBC-840(GA) _8_ YT	0.30	9.00	2.70	9.56
UBC-841(GA) _8_ YC	0.34	15.00	5.10	12.28
UBC-842 (GA) _8_ YG	0.34	15.30	5.20	15.90
UBC-855 (AC) _8_ YT	0.37	7.0	2.59	7.80
LC-46(AC) _8_ G	0.96	14.00	13.44	13.56
LC-49(TG) _6_ TCTGA	0.30	12.00	3.60	5.48
LC-6(AG) _8_ T	0.32	11.00	3.52	8.56
LC-16(CT) _8_ G	0.32	10.00	3.20	7.60
LC-14(GA) _8_	0.35	10.00	3.50	13.60
Minimum	0.20	7.00	1.80	5.48
Maximum	0.96	15.30	13.44	15.90
Average	0.36	11.35	4.28	10.25

### Performance of ISSR marker

PIC value of each primer was calculated according to [27] and the mean value of PIC for all the loci of each primer was analyzed. The range of PIC value for all the 173 polymorphic loci was 0.20–0.96 with a mean of 0.36. Of 15 primers, two of them (ISSR 9 and ISSR 11) were highly informative (PIC value > 0.5) and 12 ISSR Primers (ISSR 1, ISSR 2, ISSR 3, ISSR 4, ISSR 5, ISSR 8, ISSR 10, ISSR 12, ISSR, 13, ISSR, 14 and ISSR 15) were informative (PIC value 0.25–0.50). Only one ISSR primer (ISSR 6) was low informative (PIC value < 0.25).

The EMR value of ISSR primarily depends on the polymorphic loci [28]. In our study, the highest EMR (EMR 15.3) was observed with the primer (ISSR 9) with a mean EMR 11.35 (Table 4). To validate the effective use of the ISSR marker in finger millet, MI was calculated for each primer and the highest MI was observed with the primer ISSR 11 (13.44) and the lowest MI of the ISSR primer being ISSR 6 (1.80). The resolving power (RP) is the most important parameter to determine the discriminatory efficiency of the primer. In our study, maximum RP (15.9) was observed with ISSR 9 (Table 4). ISSR 12 was observed with the lowest RP value (5.48).

Correlation provides statistical information on the nature of association among different markers of 23 genotypes. The statistical data of correlation analysis helps to determine the level of correlation. The correlation value r defines the direction of a correlation either positively (+) or negatively (−) correlated. The correlation coefficient result revealed that the values are positively correlated except the coefficient value of − 0.0256 which was negatively correlated (Table 5). However, in ISSR markers, UBC-842 (GA) _8_ YG marker had maximum Na, Ne, I, Ho and He with mean of 3.667, 2.667, 0.921, 1.000, and 0.582. In contrast, LC-46(AC) _8_ G marker had higher value of uHe and Ho of 0.889 and 1.000 respectively.

**Table 5:**
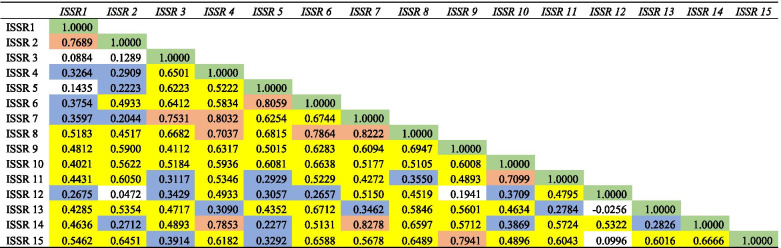
Correlation coefficient of different ISSR markers among finger millet genotypes

0.9 to 1.0 Very high positive correlation

0.7 to 0.8 High positive correlation

0.4 to 0.6Moderate positive correlation

0.2 to 0.3Low positive correlation

0.0 to < 0.3 (0.0 to − 0.3)Negligible correlation

### Genetic variation of finger millet cultivars

A high degree of genetic variation was observed among the selected cultivars of finger millet as evident from the high number of polymorphic ISSR loci. Of the 175 loci, 173 (98%) loci were polymorphic with average polymorphic loci per primer 11.50. Critical observation of our results revealed that a few of the ISSR primers who developed cultivar-specific ISSR profile polymorphic loci were cultivar specific (Table 6).

**Table 6:** Distribution of ISSR amplified fragment in finger millet varieties

Parameters	Values
	ISSR
Total number of primers screened	15
Number of primers producing polymorphism	15
Total number of loci scored	175
Total number of polymorphic loci	173
Size of amplified bands	100-2500
The average number of polymorphic loci per primer	11.50
Percentage of total loci which are polymorphic	98

### Cultivar specific ISSR primer

**Fig. 2:**
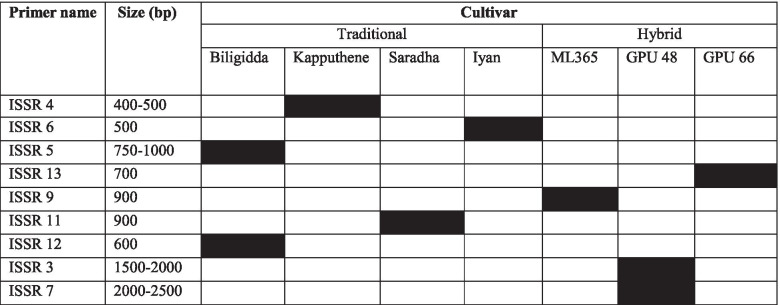
ISSR primers and their marker-specific DNA marker for identification of certain important traditional and hybrid varieties of finger millet in India

Out of 15 ISSR primers, 8 ISSR primers had developed unique cultivar/genotype-specific ISSR markers with molecular weights ranging from 400 bp to 2500 bp (Fig. 2). ISSR 4, ISSR 6, ISSR 5, ISSR 13, ISSR 11, ISSR 12, ISSR 3, and ISSR 7 had developed unique DNA bands with the molecular weights of 400–500 bp, 500 bp, 600 bp, 700 bp, 900 bp, 900–1000 bp, 1500–2000 bp, and 2000 bp–2500 bp, respectively. Although traditional and hybrid varieties of finger millets were characterized based on their floral and seed characteristics, there was a close resemblance among each other, and becoming difficult to identify [10]. Therefore, these ISSR primers could be used for precise identification of traditional and hybrid varieties of finger millets in the DNA marker-assisted breeding program of finger millet.

### Cluster analysis

**Fig. 3:**
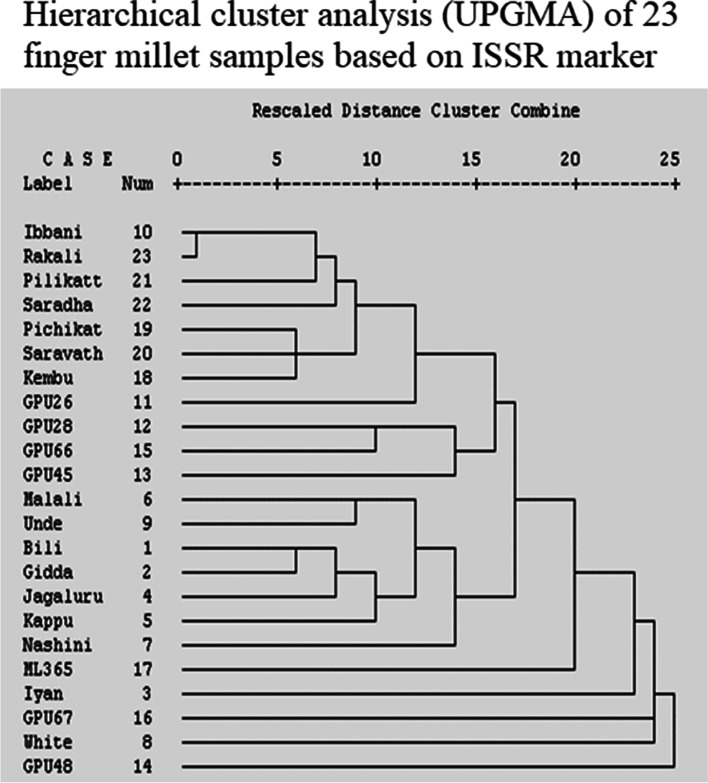
Hierarchical cluster analysis (UPGMA) of 23 finger millet samples based on ISSR marker

The dendrogram developed using UPGMA with correlation coefficient as similarity criteria revealed the following information. Out of 23 genotypes (traditional and hybrids) of finger millet. Most of the traditional varieties of Tamil Nadu province (Kembu, Pichikatty, Saravathy, Pilikatty, Saradha, Rakali sivali) formed a separate cluster while the traditional varieties of Karnataka province formed another distinct cluster. Most of the hybrids formed two distinct groups. Interestingly, both traditional and hybrid genotypes were well distanced based on their grouping pattern. This could be due to the self-breeding nature of finger millet, limiting the transfer of gene flow among the finger millets irrespective of their geographic and genetic distance (Fig. 3).

**Fig. 4:**
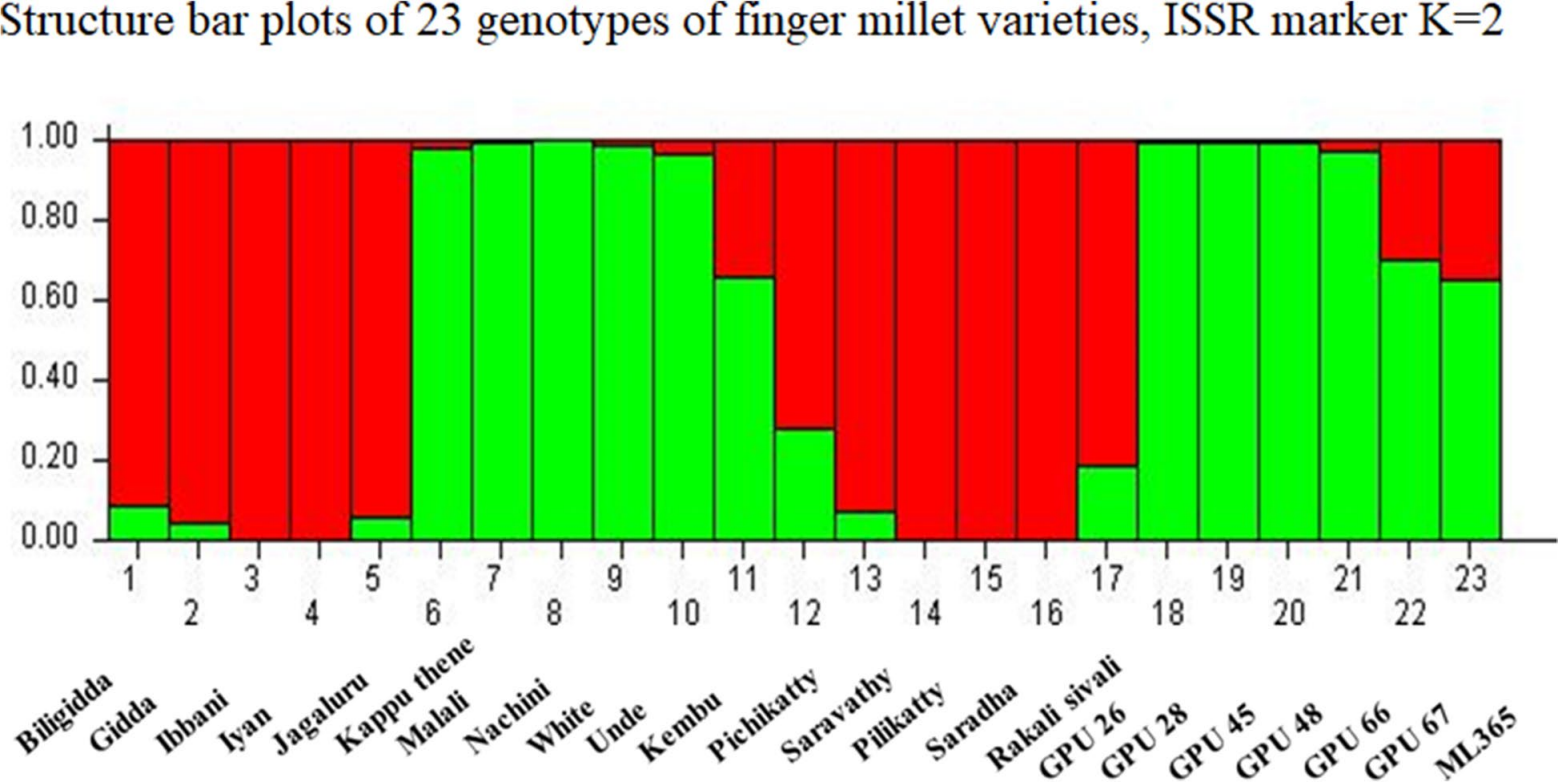
Structure bar plots of 23 genotypes of finger millet varieties, ISSR marker K=2

**Fig. 5:**
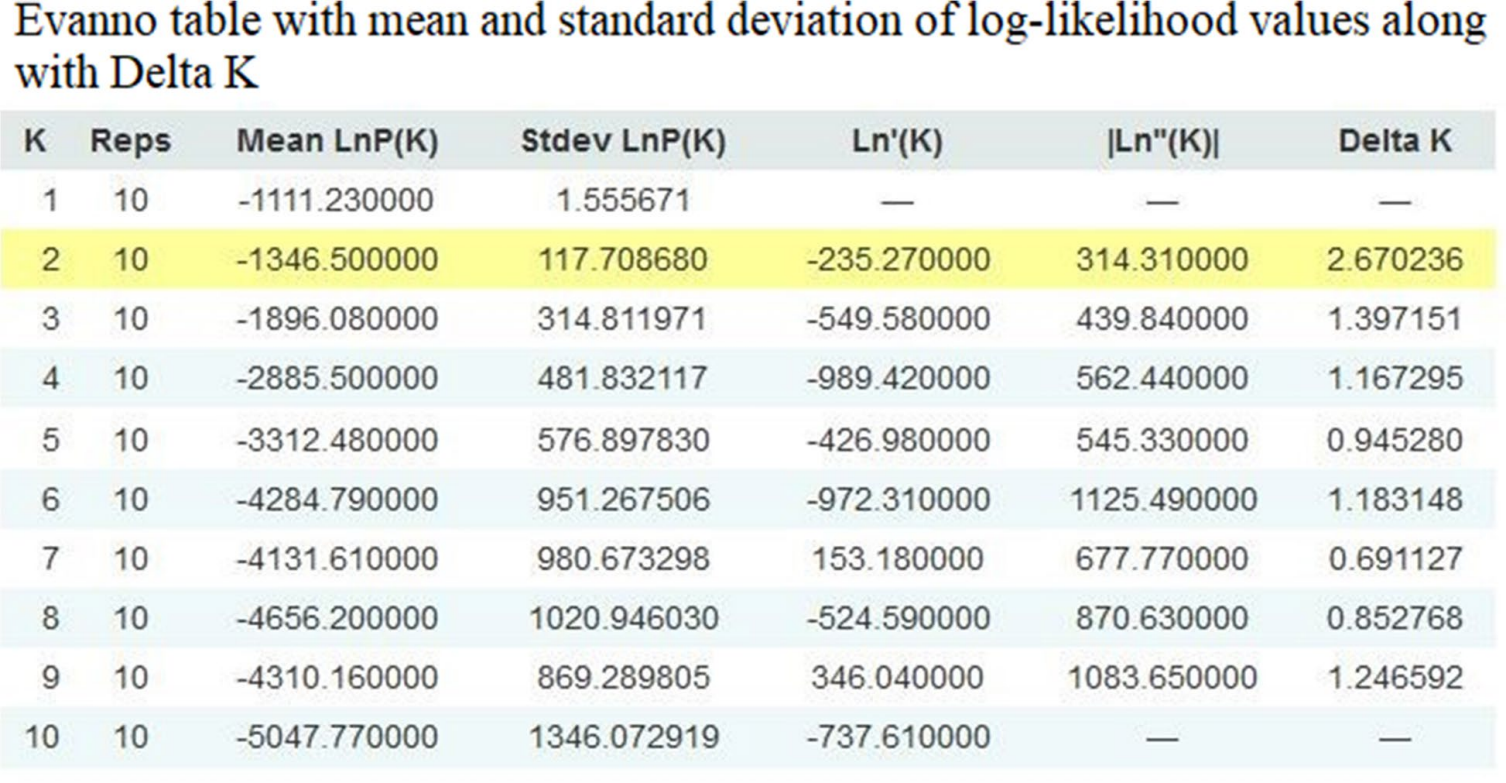
Evanno table with mean and standard deviation of log-likelihood values along with Delta K

Concerning to the genetic structure among the finger millet species, we used a non-spatial Bayesian clustering method to determine the best number of sub-populations (*K*) based on the lowest variance and highest probability of each possible number of *K*. The obtained finger millet population structure was represented in Fig. 4. The ISSR marker’s output results from STRUCTURE software 2.2.3 were analyzed using Structure harvester program which revealed that, the average estimated in probability score and lowest variance (LnP(D)), the most probable sub-population number was *K* = 2. The significant *K* value were highlighted in the evanno table (Fig. 5) which shows that *K* = 2 was significant and also supported by delta *K*. These structure analysis revealed that the samples are clustered into two main groups and undoubtedly originated from two sub-populations (groups) (Fig. 4). The first group possesses most of the traditional varieties and one hybrid variety GPU 26 which is colored in red. While the second group compresses six traditional and six hybrid varieties were coloured in green.

## Discussion

Finger millet was an agro-economically important crop that harbors drought-tolerant genes that can adapt to adverse climatic conditions [29]. Studies on finger millet genetic diversity helps in understanding the genetic resource and its genomic evolution [6]. In many previous studies, high polymorphism of ISSR marker was also reported in other plant species such as *Allium* [30], *Ocimum* [31], and *Arachis* [32]. Several studies have been carried out on genetic diversity analysis in finger millet genotypes after the establishment of molecular markers [33–35]. In finger millet, several markers were used such as RAPD, Cytochrome P450 gene-based marker, SSR [36], diversity analysis using various markers such as SSR marker [37] and RAPD, SSR, and ISSR [38–41]. The molecular marker like ISSR was found to have a high potential in determining the inter and intra-genomic diversity [10, 42]. Polymorphism was disclosed substantially by ISSR analysis [43]. In the previous report, ISSR and RAPD markers were used to assess the genetic relatedness of three-finger millet varieties with a different color [35]; 15 ISSR primers were used to determine the genetic diversity in 40 germplasms [44].

In this work, a dominant marker such as ISSR proved to be a highly efficient tool in discriminating power among the 23 varieties of finger millet. ISSR markers were selected based on robustness, higher level of polymorphism, technical simplicity, and easy adaptability to any plant species. In this study, the average PIC value of 0.36 was observed which was closer to 0.5, the maximum value for any dominant marker [45]. The high PIC value in this study reflects the efficiency of ISSR markers that are suitable for differentiating the finger millet and its closely related varieties. It was reported that resolving power (RP) can strongly correlate and distinguish between genotypes [21]. The quantitative data of RP allows comparisons between the primers which can distinguish all genotypes. In the present study, an average RP value of 10.25 proved the efficiency of the ISSR marker to discriminate among the finger millet genotypes. ISSR provides more effective information on *N*, *Na*, *Ne*, *Ho*, *He*, *uHe*, and *I* while the high relatively level in the number of effective alleles followed the pattern: UBC-842 (GA) _8_ YG > LC-46(AC) _8_ G > UBC-834(AG) _8_ YT. This finding suggested that ISSR had discrimination power for group level by genetic structure analysis by finding the best number of sub-populations (*k*). In correlation analysis, we observed a positive correlation between the ISSR markers. Here, we reported a detailed overview of genetic structure analysis based on ISSR data. Here, the dominant ISSR marker could differentiate the finger millet varieties and can identify the sub-population of finger millet.

## Conclusion

In this study, we tested with 6 traditional varieties from Tamil Nadu, 10 traditional varieties from Karnataka, and 7 hybrid varieties from Karnataka even though the samples were collected from different regions but some were having a close relationship between them was observed in the dendrogram. In the future, strategies for the development of desired genotypes can be studied based on finger millet genetic diversity. Therefore, the present study was done to analyze the genetic diversity of finger millet traditional samples with hybrid using ISSR markers. The present study has drawn the following conclusions. ISSR markers were found to be very efficient for genotyping of finger millets as these markers are generally dominant. Although a few numbers of reports are available on ISSR markers, our report is found to have useful applications in the precise identification of both traditional and hybrid cultivars of finger millets. Because finger millet is sticky self-breeding in nature, limitation of gene flow either within the traditional cultivars or between the traditional and hybrid varieties are limited. This is one of the major bottlenecks in the genetic improvement of finger millet. Thus, the present result is expected to form a foundation for molecular assisted breeding in finger millet. As finger millet germplasm has a diverse characteristic feature to nutritional value, the ISSR marker is certainly very handy in screening elite germplasm for breeding and management. Despite several reports in finger millet, this is the first report revealing cultivar-specific ISSR primers for precise identification of finger millet cultivars.

This study reveals the usefulness of additional ten more primers (ISSR 1 to ISSR 10). These primers were not used in finger millet previously. Although a very few reports on ISSR marker is published in finger millet, cultivar specific ISSR markers were not reported. Therefore, the present work is expected to have direct application in the context of DNA marker-assisted breeding in finger millet.

This is the first report of detecting genetic variation and the relationship of finger millet between traditional and hybrid varieties.

## Data Availability

All the data generated and analyzed during this study are included in this article.

## Abbreviations

ISSR: Inter Simple Sequence Repeat
PIC: Polymorphic information content
EMR: Effective multiplex ratio
MI: Marker index
RP: Resolving power
UPGMA: Unweighted pair-group method with arithmetic average
